# Supplementary material for: Changes in the gene expression profile during spontaneous migraine attacks
Source: Sci Rep. 2021 Apr 15;11:8294. doi: 10.1038/s41598-021-87503-5 (PMC8050061; doi:10.1038/s41598-021-87503-5)
Supplement: Supplementary file 1 — Supplementary Information 1. [file 41598_2021_87503_MOESM1_ESM.pdf]

Changes in the gene expression profile during spontaneous migraine attacks

Running head: RNA-sequencing of spontaneous migraine attacks

Lisette J.A. Kogelman[1*δ], Katrine Falkenberg[1*], Alfonso Buil[2], Pau Erola[3], Julie Courraud[4], Susan Svane Laursen[4], Tom Michoel[5], Jes Olesen[1], Thomas F. Hansen[1,2,6 δ].

[*]Contributed equally

[1] Danish Headache Center, Department of Neurology, Rigshospitalet Glostrup, Glostrup, Denmark.

[2] Institute for Biological Psychiatry, Mental Health Center Sct. Hans, Denmark

[3]MRC Integrative Epidemiology Unit, University of Bristol, UK

[4]Department of Clinical Biochemistry and Immunology, Statens Serum Institute Copenhagen, Copenhagen, Denmark

[5]Computational Biology Unit, Department of Informatics, University of Bergen, Norway

[6] Novo Nordic Foundation Centre for protein research, Copenhagen University, Copenhagen, Denmark

[δ]Corresponding authors: Senior Bioinformatician, Lisette J.A. Kogelman, Lisette.kogelman@regionh.dk and Assoc. Prof. Thomas Folkmann Hansen, thomas.hansen@regionh.dk

Rigshospitalet - Neurocentret - Glostrup, Nordstjernevej 42, 2600 Glostrup, Denmark

Supplementary Materials

Table S1. Expression of migraine GWAS genes during and outside migraine attack.

Table S2. Enriched migraine-specific GO-terms.

Table S1. Expression of migraine GWAS genes during and outside migraine attack.

	Migraine attack (A)	*After treatment (B)*	*Outside attack (C)*	*After cold pressor test (D)*
ADAMTSL4	13385	13586	13196	14529
ASTN2	294	292	290	349
CARF	713	676	713	916
CCM2L	18	18	17	21
CFDP1	1476	1296	1332	1528
DOCK4	953	947	956	1107
ECM1	110	103	103	103
HCK	64250	59483	62189	62629
HEY2	18	15	19	16
HJURP	141	136	141	154
HPSE2	9	10	10	10
HTRA1	64	51	59	61
IGSF9B	416	411	452	497
IMMP2L	145	132	143	155
ITPK1	26348	24118	24951	24431
JAG1	232	252	255	301
KCNK5	85	81	91	86
LRP1	16558	14068	15993	15534
MED14	3278	3135	3227	3649
MEF2D	12061	11928	12267	12732
MPPED2	92	91	99	112
NCOA7	1632	1562	1637	1975
NLRP1	59740	57036	57938	62130
NOTCH4	531	456	517	523
NRP1	375	349	394	405
PHACTR1	1845	1978	1702	1877
PLCE1	581	588	607	725
PRDM16	38	38	40	44
REST	3182	3144	3213	3858
RNF213	80118	77507	81327	85673
SLC24A3	176	186	170	174
SPINK2	30	30	30	35
STAT6	86242	81865	84292	86766
TGFBR2	26516	26105	26301	30033
TRPM8	100	96	97	114
TSPAN2	1938	2027	1984	2466
UFL1	1286	1217	1282	1691
USP9X	10365	10203	9859	11268
WSCD1	12	11	12	12
YAP1	18	18	16	22
ZCCHC14	1297	1244	1298	1423

Table S2. Enriched migraine-specific GO-terms.

Network Cluster[*]	*cluster*	*GOterm*[**]	*process*	*adjP*[***]
A-0	0	GO:0000079	regulation of cyclin-dependent protein serine/threonine kinase activity	0.0148
A-0	0	GO:0006323	DNA packaging	0.00988
A-0	0	GO:0007018	microtubule-based movement	0.0332
A-0	0	GO:0007063	regulation of sister chromatid cohesion	0.0177
A-0	0	GO:0018105	peptidyl-serine phosphorylation	0.0305
A-0	0	GO:0030330	DNA damage response, signal transduction by p53 class mediator	0.0474
A-0	0	GO:0030890	positive regulation of B cell proliferation	0.0246
A-0	0	GO:0031571	mitotic G1 DNA damage checkpoint	0.0294
A-0	0	GO:0032466	negative regulation of cytokinesis	0.0294
A-0	0	GO:0042770	signal transduction in response to DNA damage	0.0102
A-0	0	GO:0044783	G1 DNA damage checkpoint	0.0294
A-0	0	GO:0044819	mitotic G1/S transition checkpoint	0.0294
A-0	0	GO:0045740	positive regulation of DNA replication	0.00203
A-0	0	GO:0048518	positive regulation of biological process	0.0102
A-0	0	GO:0050871	positive regulation of B cell activation	0.0474
A-0	0	GO:0051098	regulation of binding	0.0299
A-0	0	GO:0051302	regulation of cell division	0.0408
A-0	0	GO:0051782	negative regulation of cell division	0.0177
A-0	0	GO:0071158	positive regulation of cell cycle arrest	0.0299
A-0	0	GO:0071163	DNA replication preinitiation complex assembly	0.00458
A-0	0	GO:0072413	signal transduction involved in mitotic cell cycle checkpoint	0.0246
A-0	0	GO:0072431	signal transduction involved in mitotic G1 DNA damage checkpoint	0.0246
A-0	0	GO:0090329	regulation of DNA-dependent DNA replication	0.0019
A-0	0	GO:1902400	intracellular signal transduction involved in G1 DNA damage checkpoint	0.0246
A-0	0	GO:1902402	signal transduction involved in mitotic DNA damage checkpoint	0.0246
A-0	0	GO:1902403	signal transduction involved in mitotic DNA integrity checkpoint	0.0246
A-0	0	GO:1902806	regulation of cell cycle G1/S phase transition	0.0072
A-0	0	GO:1904029	regulation of cyclin-dependent protein kinase activity	0.0224
A-0	0	GO:1904668	positive regulation of ubiquitin protein ligase activity	0.0294
A-0	0	GO:2000045	regulation of G1/S transition of mitotic cell cycle	0.0135
A-0	0	GO:2000105	positive regulation of DNA-dependent DNA replication	0.000309
A-1	1	GO:0006266	DNA ligation	0.0146
A-1	1	GO:0017148	negative regulation of translation	0.0349
A-1	1	GO:0031124	mRNA 3'-end processing	0.0146
A-1	1	GO:0031392	regulation of prostaglandin biosynthetic process	0.0146

A-1	1	GO:0032069	regulation of nuclease activity	0.00411
A-1	1	GO:0035722	interleukin-12-mediated signaling pathway	0.0393
A-1	1	GO:0070671	response to interleukin-12	0.0467
A-1	1	GO:0071349	cellular response to interleukin-12	0.0467
A-1	1	GO:0090305	nucleic acid phosphodiester bond hydrolysis	0.0118
A-1	1	GO:2001279	regulation of unsaturated fatty acid biosynthetic process	0.0264
A-2	2	GO:0001819	positive regulation of cytokine production	0.0314
A-2	2	GO:0002281	macrophage activation involved in immune response	0.0212
A-2	2	GO:0008625	extrinsic apoptotic signaling pathway via death domain receptors	0.00974
A-2	2	GO:0019370	leukotriene biosynthetic process	0.0212
A-2	2	GO:0032102	negative regulation of response to external stimulus	0.0123
A-2	2	GO:0032271	regulation of protein polymerization	0.0314
A-2	2	GO:0032479	regulation of type I interferon production	0.0189
A-2	2	GO:0032648	regulation of interferon-beta production	0.00642
A-2	2	GO:0032649	regulation of interferon-gamma production	0.0367
A-2	2	GO:0032652	regulation of interleukin-1 production	0.0426
A-2	2	GO:0032677	regulation of interleukin-8 production	0.0468
A-2	2	GO:0032688	negative regulation of interferon-beta production	0.0212
A-2	2	GO:0070887	cellular response to chemical stimulus	0.0314
A-2	2	GO:0071345	cellular response to cytokine stimulus	0.0204
A-3	3	GO:0000186	activation of MAPKK activity	0.0399
A-3	3	GO:0002224	toll-like receptor signaling pathway	0.0177
A-3	3	GO:0002755	MyD88-dependent toll-like receptor signaling pathway	0.0114
A-3	3	GO:0007049	cell cycle	0.0149
A-3	3	GO:0007249	I-kappaB kinase/NF-kappaB signaling	0.0341
A-3	3	GO:0007257	activation of JUN kinase activity	0.0436
A-3	3	GO:0009892	negative regulation of metabolic process	0.0443
A-3	3	GO:0009893	positive regulation of metabolic process	0.0377
A-3	3	GO:0009896	positive regulation of catabolic process	0.0393
A-3	3	GO:0010556	regulation of macromolecule biosynthetic process	0.0207
A-3	3	GO:0010604	positive regulation of macromolecule metabolic process	0.0144
A-3	3	GO:0016579	protein deubiquitination	0.0406
A-3	3	GO:0019222	regulation of metabolic process	0.0335
A-3	3	GO:0030334	regulation of cell migration	0.0389
A-3	3	GO:0031058	positive regulation of histone modification	0.0304
A-3	3	GO:0031063	regulation of histone deacetylation	0.021
A-3	3	GO:0031065	positive regulation of histone deacetylation	0.0145
A-3	3	GO:0031323	regulation of cellular metabolic process	0.00704
A-3	3	GO:0031324	negative regulation of cellular metabolic process	0.0335
A-3	3	GO:0031325	positive regulation of cellular metabolic process	0.0287
A-3	3	GO:0031326	regulation of cellular biosynthetic process	0.0177
A-3	3	GO:0031399	regulation of protein modification process	0.0287

A-3	3	GO:0031401	positive regulation of protein modification process	0.0393
A-3	3	GO:0032268	regulation of cellular protein metabolic process	0.0281
A-3	3	GO:0033554	cellular response to stress	0.0399
A-3	3	GO:0035556	intracellular signal transduction	0.0389
A-3	3	GO:0038089	positive regulation of cell migration by vascular endothelial growth factor signaling pathway	0.0393
A-3	3	GO:0042176	regulation of protein catabolic process	0.0324
A-3	3	GO:0044264	cellular polysaccharide metabolic process	0.0399
A-3	3	GO:0045732	positive regulation of protein catabolic process	0.0346
A-3	3	GO:0048523	negative regulation of cellular process	0.0315
A-3	3	GO:0051128	regulation of cellular component organization	0.021
A-3	3	GO:0051171	regulation of nitrogen compound metabolic process	0.0065
A-3	3	GO:0051173	positive regulation of nitrogen compound metabolic process	0.0177
A-3	3	GO:0051270	regulation of cellular component movement	0.0242
A-3	3	GO:0051338	regulation of transferase activity	0.0287
A-3	3	GO:0060255	regulation of macromolecule metabolic process	0.0106
A-3	3	GO:0061136	regulation of proteasomal protein catabolic process	0.00915
A-3	3	GO:0080135	regulation of cellular response to stress	0.021
A-3	3	GO:0090045	positive regulation of deacetylase activity	0.0242
A-3	3	GO:0090312	positive regulation of protein deacetylation	0.021
A-3	3	GO:0150065	regulation of deacetylase activity	0.0242
A-3	3	GO:1901673	regulation of mitotic spindle assembly	0.0348
A-3	3	GO:1901699	cellular response to nitrogen compound	0.0315
A-3	3	GO:1901725	regulation of histone deacetylase activity	0.0242
A-3	3	GO:1901727	positive regulation of histone deacetylase activity	0.0242
A-3	3	GO:1901800	positive regulation of proteasomal protein catabolic process	0.0335
A-3	3	GO:1903362	regulation of cellular protein catabolic process	0.0389
A-3	3	GO:2000112	regulation of cellular macromolecule biosynthetic process	0.0287
A-3	3	GO:2000145	regulation of cell motility	0.0214
A-4	4	GO:0070527	platelet aggregation	0.043
A-9	9	GO:0003002	regionalization	0.0359
A-9	9	GO:0003008	system process	0.0233
A-9	9	GO:0021513	spinal cord dorsal/ventral patterning	0.0374
A-9	9	GO:0030325	adrenal gland development	0.0339
A-9	9	GO:0050877	nervous system process	0.0145
A-9	9	GO:0050906	detection of stimulus involved in sensory perception	0.033
A-10	10	GO:0006511	ubiquitin-dependent protein catabolic process	0.0405
A-10	10	GO:0019941	modification-dependent protein catabolic process	0.0432
A-10	10	GO:0043161	proteasome-mediated ubiquitin-dependent protein catabolic process	0.016
A-10	10	GO:0043412	macromolecule modification	0.0298
A-10	10	GO:0043632	modification-dependent macromolecule catabolic process	0.0462

A-10	10	GO:0051603	proteolysis involved in cellular protein catabolic process	0.0392
A-12	12	GO:0001932	regulation of protein phosphorylation	0.0444
A-12	12	GO:0002698	negative regulation of immune effector process	0.0186
A-12	12	GO:0002920	regulation of humoral immune response	0.0354
A-12	12	GO:0003013	circulatory system process	0.0302
A-12	12	GO:0007154	cell communication	0.0243
A-12	12	GO:0007165	signal transduction	0.0369
A-12	12	GO:0007166	cell surface receptor signaling pathway	0.00329
A-12	12	GO:0008015	blood circulation	0.0254
A-12	12	GO:0009967	positive regulation of signal transduction	0.0186
A-12	12	GO:0023052	signaling	0.0398
A-12	12	GO:0031348	negative regulation of defense response	0.00161
A-12	12	GO:0033993	response to lipid	0.0428
A-12	12	GO:0034367	protein-containing complex remodeling	0.00412
A-12	12	GO:0034368	protein-lipid complex remodeling	0.00412
A-12	12	GO:0034369	plasma lipoprotein particle remodeling	0.00412
A-12	12	GO:0034370	triglyceride-rich lipoprotein particle remodeling	0.00959
A-12	12	GO:0034372	very-low-density lipoprotein particle remodeling	0.00419
A-12	12	GO:0045926	negative regulation of growth	0.0159
A-12	12	GO:0050790	regulation of catalytic activity	0.0385
A-12	12	GO:0050900	leukocyte migration	0.0263
A-12	12	GO:0051239	regulation of multicellular organismal process	0.0475
A-12	12	GO:0051241	negative regulation of multicellular organismal process	0.00863
A-12	12	GO:0051336	regulation of hydrolase activity	0.0126
A-12	12	GO:0051716	cellular response to stimulus	0.0339
A-12	12	GO:0071825	protein-lipid complex subunit organization	0.0143
A-12	12	GO:0071827	plasma lipoprotein particle organization	0.0119
A-13	13	GO:0006811	ion transport	0.00506
A-13	13	GO:0006812	cation transport	0.00394
A-13	13	GO:0034220	ion transmembrane transport	0.00183
A-13	13	GO:0098655	cation transmembrane transport	0.0237
A-13	13	GO:0098660	inorganic ion transmembrane transport	0.00853
A-14	14	GO:0002475	antigen processing and presentation via MHC class Ib	0.0364
A-14	14	GO:0006783	heme biosynthetic process	0.0301
A-14	14	GO:0010506	regulation of autophagy	0.036
A-14	14	GO:0019724	B cell mediated immunity	0.0418
A-14	14	GO:0030097	hemopoiesis	0.0205
A-14	14	GO:0042592	homeostatic process	0.0192
A-14	14	GO:0048518	positive regulation of biological process	0.0299
A-14	14	GO:0048534	hematopoietic or lymphoid organ development	0.036
A-14	14	GO:0048878	chemical homeostasis	0.036
A-14	14	GO:0050896	response to stimulus	0.016
A-16	16	GO:0010035	response to inorganic substance	0.0304
A-16	16	GO:0010038	response to metal ion	0.0497

A-16	16	GO:0051249	regulation of lymphocyte activation	0.049
A-17	17	GO:0001655	urogenital system development	0.0431
A-17	17	GO:0072001	renal system development	0.045
A-19	19	GO:0006464	cellular protein modification process	0.0435
A-19	19	GO:0006879	cellular iron ion homeostasis	0.0286
A-19	19	GO:0036211	protein modification process	0.0435
A-19	19	GO:0045638	negative regulation of myeloid cell differentiation	0.0344
B-0	0	GO:0033260	nuclear DNA replication	0.0105
B-0	0	GO:0035088	establishment or maintenance of apical/basal cell polarity	0.0474
B-0	0	GO:0042074	cell migration involved in gastrulation	0.0105
B-0	0	GO:0044786	cell cycle DNA replication	0.0105
B-0	0	GO:0051307	meiotic chromosome separation	0.0172
B-0	0	GO:0061245	establishment or maintenance of bipolar cell polarity	0.0474
B-1	1	GO:0000209	protein polyubiquitination	0.00132
B-1	1	GO:0002220	innate immune response activating cell surface receptor signaling pathway	0.03
B-1	1	GO:0002283	neutrophil activation involved in immune response	0.0491
B-1	1	GO:0002478	antigen processing and presentation of exogenous peptide antigen	0.0218
B-1	1	GO:0006511	ubiquitin-dependent protein catabolic process	0.0292
B-1	1	GO:0010498	proteasomal protein catabolic process	0.024
B-1	1	GO:0019884	antigen processing and presentation of exogenous antigen	0.03
B-1	1	GO:0019941	modification-dependent protein catabolic process	0.0301
B-1	1	GO:0033209	tumor necrosis factor-mediated signaling pathway	0.0216
B-1	1	GO:0035567	non-canonical Wnt signaling pathway	0.0485
B-1	1	GO:0043161	proteasome-mediated ubiquitin-dependent protein catabolic process	0.0127
B-1	1	GO:0043312	neutrophil degranulation	0.047
B-1	1	GO:0043632	modification-dependent macromolecule catabolic process	0.0319
B-1	1	GO:0048002	antigen processing and presentation of peptide antigen	0.0347
B-1	1	GO:0051603	proteolysis involved in cellular protein catabolic process	0.0395
B-1	1	GO:0051973	positive regulation of telomerase activity	0.0485
B-1	1	GO:0071824	protein-DNA complex subunit organization	0.0485
B-1	1	GO:0090090	negative regulation of canonical Wnt signaling pathway	0.0496
B-1	1	GO:1901987	regulation of cell cycle phase transition	0.0142
B-1	1	GO:1901990	regulation of mitotic cell cycle phase transition	0.0445
B-1	1	GO:2000736	regulation of stem cell differentiation	0.0202
B-2	2	GO:0006333	chromatin assembly or disassembly	0.0157
B-2	2	GO:0006334	nucleosome assembly	0.0168
B-2	2	GO:0009611	response to wounding	0.00663
B-2	2	GO:0030041	actin filament polymerization	0.0344
B-2	2	GO:0031497	chromatin assembly	0.0318

B-2	2	GO:0034728	nucleosome organization	0.0168
B-2	2	GO:0043313	regulation of neutrophil degranulation	0.0169
B-2	2	GO:0043933	protein-containing complex subunit organization	0.00292
B-2	2	GO:0051345	positive regulation of hydrolase activity	0.0327
B-2	2	GO:0051651	maintenance of location in cell	0.0225
B-2	2	GO:0065003	protein-containing complex assembly	0.00503
B-2	2	GO:0065004	protein-DNA complex assembly	0.0268
B-2	2	GO:0071824	protein-DNA complex subunit organization	0.0269
B-2	2	GO:0090200	positive regulation of release of cytochrome c from mitochondria	0.0277
B-2	2	GO:0097435	supramolecular fiber organization	0.0426
B-2	2	GO:2001235	positive regulation of apoptotic signaling pathway	0.0344
B-2	2	GO:2001242	regulation of intrinsic apoptotic signaling pathway	0.0104
B-2	2	GO:2001243	negative regulation of intrinsic apoptotic signaling pathway	0.0426
B-3	3	GO:0006643	membrane lipid metabolic process	0.0336
B-3	3	GO:0008610	lipid biosynthetic process	0.0423
B-4	4	GO:0006950	response to stress	0.00445
B-9	9	GO:0001708	cell fate specification	0.0313
B-9	9	GO:0003156	regulation of animal organ formation	0.0356
B-9	9	GO:0009653	anatomical structure morphogenesis	0.0444
B-9	9	GO:0009887	animal organ morphogenesis	0.0464
B-9	9	GO:0021536	diencephalon development	0.029
B-9	9	GO:0022612	gland morphogenesis	0.014
B-9	9	GO:0030154	cell differentiation	0.04
B-9	9	GO:0030539	male genitalia development	0.029
B-9	9	GO:0030850	prostate gland development	0.0464
B-9	9	GO:0048468	cell development	0.0256
B-9	9	GO:0048598	embryonic morphogenesis	0.0356
B-9	9	GO:0048646	anatomical structure formation involved in morphogenesis	0.0464
B-9	9	GO:0048665	neuron fate specification	0.014
B-9	9	GO:0048732	gland development	0.0225
B-9	9	GO:0048736	appendage development	0.0444
B-9	9	GO:0048806	genitalia development	0.0178
B-9	9	GO:0048863	stem cell differentiation	0.0356
B-9	9	GO:0048869	cellular developmental process	0.0444
B-9	9	GO:0060173	limb development	0.0444
B-9	9	GO:0060571	morphogenesis of an epithelial fold	0.0319
B-9	9	GO:2000036	regulation of stem cell population maintenance	0.0356
B-10	10	GO:0036092	phosphatidylinositol-3-phosphate biosynthetic process	0.021
B-11	11	GO:0070098	chemokine-mediated signaling pathway	0.0306
B-11	11	GO:0140131	positive regulation of lymphocyte chemotaxis	0.0143
B-11	11	GO:1990868	response to chemokine	0.0399
B-11	11	GO:1990869	cellular response to chemokine	0.0399

B-11	11	GO:2000503	positive regulation of natural killer cell chemotaxis	0.00757
B-12	12	GO:0022617	extracellular matrix disassembly	0.0412
B-12	12	GO:0061025	membrane fusion	0.00803
B-13	13	GO:0001508	action potential	0.0124
B-13	13	GO:0009698	phenylpropanoid metabolic process	0.0153
B-13	13	GO:0009804	coumarin metabolic process	0.0153
B-14	14	GO:0002479	antigen processing and presentation of exogenous peptide antigen via MHC class I, TAP-dependent	0.0304
B-14	14	GO:0006897	endocytosis	0.0357
B-14	14	GO:0034383	low-density lipoprotein particle clearance	0.00807
B-14	14	GO:0043277	apoptotic cell clearance	0.0485
B-14	14	GO:1901685	glutathione derivative metabolic process	0.0493
B-14	14	GO:1901687	glutathione derivative biosynthetic process	0.0493
B-16	16	GO:0097428	protein maturation by iron-sulfur cluster transfer	0.00624
B-16	16	GO:1903578	regulation of ATP metabolic process	0.0371
B-17	17	GO:0019752	carboxylic acid metabolic process	0.0323
B-17	17	GO:0042537	benzene-containing compound metabolic process	0.00297
B-17	17	GO:0048519	negative regulation of biological process	0.0462
B-17	17	GO:0072676	lymphocyte migration	0.0384
B-19	19	GO:0002221	pattern recognition receptor signaling pathway	0.00767
B-19	19	GO:0002755	MyD88-dependent toll-like receptor signaling pathway	0.0421
B-19	19	GO:0006954	inflammatory response	0.0232
B-19	19	GO:0007249	I-kappaB kinase/NF-kappaB signaling	0.0439
B-19	19	GO:0009617	response to bacterium	0.0343
B-19	19	GO:0016458	gene silencing	0.0439
B-19	19	GO:0032728	positive regulation of interferon-beta production	0.0419
B-19	19	GO:1901222	regulation of NIK/NF-kappaB signaling	0.0368
B-19	19	GO:1904645	response to amyloid-beta	0.0421
B-19	19	GO:1904646	cellular response to amyloid-beta	0.0214

[*]Clusters are marked with A and B, representing the networks 'during migraine attack' (A) and 'after treatment' (B).

[**]GOterm = Gene Ontology term

[***]P_{adj} = P-value adjusted for multiple-testing
